# Dressing a Nonpolarizable Force Field for OH^–^ in TIP4P/2005 Aqueous Solutions with Corrected Hirshfeld Charges

**DOI:** 10.1021/acs.jpclett.4c02261

**Published:** 2024-09-09

**Authors:** Marcos de Lucas, Samuel Blazquez, Jacobo Troncoso, Carlos Vega, Francisco Gámez

**Affiliations:** † Departamento de Química Física I, Fac. Ciencias Químicas, Universidad Complutense de Madrid, 28040 Madrid, España; ‡ Departamento de Física Aplicada, Universidade de Vigo, Escola de Enxeñaría Aeronaútica e do Espazo, E 32004, Ourense, España

## Abstract

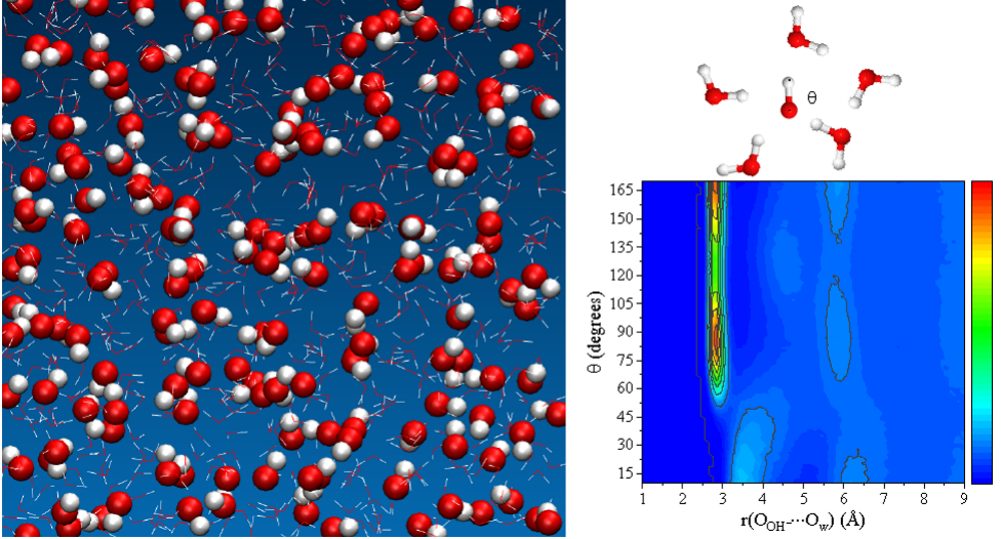

We present a rigid model for the OH^–^ ion parametrized for binary mixtures with TIP4P/2005-type water molecules. Li^+^, Na^+^ and K^+^ were selected as counterions, hence mimicking the important and widely used solutions of soluble alkaline hydroxides. The optimized atomic charge distributions were obtained by scaling in a factor of 0.85 those derived from the atomic dipole corrected Hirshfeld approach. The agreement between experimental and Molecular Dynamics simulation results is remarkable for a set of properties, namely, the dependence of the density of the solutions on the hydroxide concentration and on temperature, the structure (i.e., positions of the atom-to-atom radial distribution functions and coordination numbers), the viscosity coefficients, the surface tension, or the freezing point depression. The proposed optimized potential parameters for OH^–^ thus enlarge the set of models comprised within the Madrid–2019 force field and widen the potential applicability of the TIP4P/2005 water model in basic environments.

The pH value is crucial in the course of surface-mediated phenomena in soft matter^1^ and in electrochemical processes of technological relevance that are in the line of fire of *Green Chemistry*.^2^ Consequently, the availability of accurate, robust, and computationally inexpensive force fields able to grasp the main properties of water at different pH values from molecular simulations is urgent for chemical and technological applications. Nevertheless, filling this gap is a coveted yet cumbersome task since it involves a solid force field not only for water but also for the dissolved species at play, which is particularly tricky for the ions arising from the self-ionization of water, i.e., oxonium, H_3_O^+^, and hydroxide, OH^–^, ions.

On the one hand, the development of an “all purpose” force field for bulk water constitutes a demanding exercise because of the complex scenario provided by their anomalous physicochemical features.^3^ Among the ensemble of prototypical nonpolarizable rigid models for water, the TIP4P/2005 force field stands out because it notably reproduces most thermodynamic and dynamic properties of water with remarkable accuracy,^3^ and it will be the one selected here. On the other hand, a force field for specific electrolytes is required for a given water model. The design of a force field for OH^–^ should provide the description of its geometry and structural and peculiar coordination features, as well as thermodynamic and transport properties. Particularly, diffraction^4^ and spectroscopy^5,6^ experiments, static quantum chemistry methods^7^ and *ab initio* Molecular Dynamics^8,9^ demonstrated that a dynamic 4-fold hypercoordinated Eigen-like anion (H_9_O_5_^–^) is the key OH^–^(*aq.*) motif^10^ controlling the so-called structural diffusion properties via a concentration- and temperature-dependent presolvated state. Unlike the Grotthuss mechanism in H_3_O^+^, proton transfer in OH^–^ solutions demands an intermediate structural transition from a 4-fold to a 3-fold coordination, thus inducing a “proton-hole” to migrate through the solvent.^9,12^ Some new insights into this dependence have been obtained in ref (13) using a multidimensional neural network-derived potential.

In this context, although resigned to not describe the H-breaking mediated contribution to the OH^–^ diffusion, some classical force fields were successfully developed because the diffusion mechanism does not modify the average bulk structure.^12^ These models range from spherical^14−17,21^ to multisite^18,19^ to charge-ring^20^ distributions.

Here, the spirit of the Madrid-2019 force field for ions is followed^22,23^ for constructing a force field for OH^–^ whose results will be confronted against experimental thermodynamic, dynamic, interfacial and structural data. However, as pointed out by some of us, fitted charges might reproduce the potential energy surface but not the properties derived from the dipole moment surface.^24^ Therefore, atomic charges derived from recursive optimizations are not expected to be directly comparable with those coming from *first principle* calculations. Quantum-derived charges have a marked dependence on the size of the basis set onto which the wave function is projected. Here, to relieve this sizable effect, we carried out a population analysis of the isolated OH^–^ ion in the framework of the atomic dipole corrected Hirshfeld approach (ADCH)^25^ at the B3LYP/6-311++G(d,p) level, from which the atomic charges are obtained as *q*_*O*_ = −1.262*e* and *q*_*H*_ = +0.262*e* for the O and H atoms, respectively (see Methods). These charges are, in absolute values, higher than those reported in ref (26) from the Atoms in Molecules approach for *q*_*O*_ (−1.050*e* and −0.975*e*) and in between for *q*_*H*_ (+0.300*e* and +0.225*e*). On top of that, the Madrid-2019 force field for electrolytes (inspired by the Electronic Continuum Correction^27^) uses noninteger ion charges (*q*) of ±0.85*e* to implicitly incorporate the Coulombic screening effect of the electrons (i.e., the high frequency component of the dielectric constant) on the interionic interactions. Although some improvements in the description of the transport properties have been reported when a charge of ±0.75*e* is chosen,^28^ the selection of the Madrid-2019 force field can be considered as a compromise solution for modeling ions that reasonably capture the bulk of the properties of electrolytic solutions.^28^ The situation is somehow more complicated when dealing with polyatomic species, since the distribution of point charges within the molecule constitutes an additional degree of freedom that plays an important role in the design of a force field, as will be discussed below.

The proposed model is then constructed for these three salts by considering a Lennard-Jones interaction, characterized by a set of interatomic length (σ_*ij*_) and energy (ε_*ij*_) parameters, and a Coulombic interaction described by the aforementioned set of atomic charges {*q*_i_} obtained from the ADCH approach. Lennard-Jones parameters were fitted to match the experimental–computational agreement of the density–composition curve (see the detailed procedure in refs (22 and 23)). The whole set of optimized parameters is shown in Table 1. The self- and cross-Lennard-Jones parameters and atomic charges of the counterions and water were also taken from the Madrid-2019 force field.^22,23^ Briefly, the ion–water parameters were fitted to reproduce the experimental densities of the aqueous solutions over the entire concentration range. The ion–ion interactions were then adjusted to avoid precipitation of the salt and to fine-tune the densities at high concentrations (below the solubility limit). Once the force field was optimized, we performed an intensive simulation survey to extract structural, thermodynamics and transport properties of basic watery solutions (see Methods). In Figure 1(a)–(b) we present the results for both the density and viscosity of these salts as a function of the molality (i.e., number of moles of solute per kilogram of solvent) in comparison with both experimental and simulation data evaluated with an optimized model with total charges scaled to ±0.75*e* reported in ref (26) (model H hereinafter). Notice that NaOH and KOH are highly soluble in water at room temperature (with solubilities of ∼25 *m* and ∼20 *m*, respectively), whereas the solubility of LiOH is significantly smaller (∼5 *m*). As mentioned above, it is expected that a model with a net charge of 0.75*e* was able to reproduce the transport properties more accurately than that with a charge of 0.85*e*. For comparative purposes, the relative average deviations of the *N* computed data have been defined as *s* = 1/*N*∑_*i*_|*X*_*i*_ – *x*_*i*_|/*X*_*i*_, with *x*_*i*_ the simulated data at a given *m* and *X*_*i*_ the corresponding value from the fit of the experimental data. The densities are better predicted by the presented model, as observed in Figure 1(a). Particularly, for KOH, *s* increases from 0.3% in our model to 0.6% for the model H. The improvement is much more notable for NaOH, with a factor of ∼10 between the here obtained force field (*s* = 0.15%) and that obtained for the model H. Only at high concentrations (>10 *m*) are the experimental values slightly underestimated. For instance, at 16 *m*, the deviation of the density is 3% for KOH and ∼6% for NaOH. Nevertheless, even when the model H is preferable if the specific purpose is a quantitative evaluation of the viscosity coefficients, the predictions of the viscosity coefficients are improved with respect to the tentative model with the same global charge (*q* = ±0.85*e*) proposed in ref (26). Such an effect points out the importance of both the method selected for assigning partial atomic charges and also the charge distribution when dealing with polyatomic ions. Overall, it is shown that the Madrid-2019 force field dressed with an ADCH charge distribution grasped the viscosity and bulk densities of basic solutions, despite specific modifications that might result in an enhancement of the agreement of a certain property (for instance, viscosity coefficients). However, as expected from a classical (nonreactive) model, the calculated diffusion coefficient at infinite dilution of the OH^–^ ion (1.3 × 10^–9^ m^2^·s^–1^, as evaluated by extrapolating the calculated diffusion coefficients to *m* → 0) is 4 times smaller than those obtained experimentally from the determination of the limiting molar conductivities (5.3 × 10^–9^ m^2^·s^–1^, see ref (30)), provided classical Molecular Dynamics captures only the (nonreactive) Brownian contribution to the diffusion coefficient.

**Table 1:** Coulombic and Lennard-Jones Parameters of the OH^–^ Force Field, as Obtained in This Work^a^

Self-interaction parameters		
Parameter	Value	Source
*q*_O_	–1.0727*e*	This work
*q*_H_	0.2227*e*	This work
ε_*O*_OH_–*O*_OH__/*k*_*B*_	30.1753	From ref (26)
ε_*H*_OH_–*H*_OH__/*k*_*B*_	22.1192	From ref (26)
σ_*O*_OH_–*O*_OH__	3.4000000	This work
σ_*H*_OH_–*H*_OH__	1.4430000	From ref (26)

Cross-interaction parameters		
Parameter	Value	Mixing route
ε_*O*_OH_–*H*_OH__/*k*_*B*_	25.8351	LB
ε_*O*_OH_–*O*_*w*__/*k*_*B*_	53.0312	LB
ε_*O*_OH_–Li^+^_/*k*_*B*_	39.7374	LB
ε_*O*_OH_–Na^+^_/*k*_*B*_	73.0998	LB
ε_*O*_OH_–K^+^_/*k*_*B*_	84.8904	LB
ε_*H*_OH_–*O*_*w*__/*k*_*B*_	45.4040	LB
ε_*H*_OH_–Li^+^_/*k*_*B*_	34.0219	LB
ε_*H*_OH_–Na^+^_/*k*_*B*_	62.5858	LB
ε_*H*_OH_–K^+^_/*k*_*B*_	72.6826	LB
σ_*O*_OH_–*H*_OH__	2.4215000	LB
σ_*O*_OH_–*O*_*w*__	3.4500000	n-LB (∼5%)
σ_*O*_OH_–Li^+^_	2.6448500	n-LB (∼9%)
σ_*O*_OH_–Na^+^_	3.0336834	n-LB (∼7%)
σ_*O*_OH_–K^+^_	3.2000000	n-LB (∼11%)
σ_*H*_OH_–*O*_*w*__	2.3009500	LB
σ_*H*_OH_–Li^+^_	1.4413500	LB
σ_*H*_OH_–Na^+^_	1.8301834	LB
σ_*H*_OH_–K^+^_	1.8722000	LB

a Partial charges *q*_H_ = 0.2227*e*, *q*_O_ = −1.0727*e* were calculated by scaling those obtained by means of the ADCH population method. The O–H bond distance was set to 0.98 Å. Values of σ_*ij*_ and ε_*ij*_/k_B_ are given in units of Å and K, respectively. We specified whether the Lorentz–Berthelot rule is followed (LB) or not (n-LB). In the latter case, the deviations from the LB rule are indicated within brackets.

**Figure 1:**
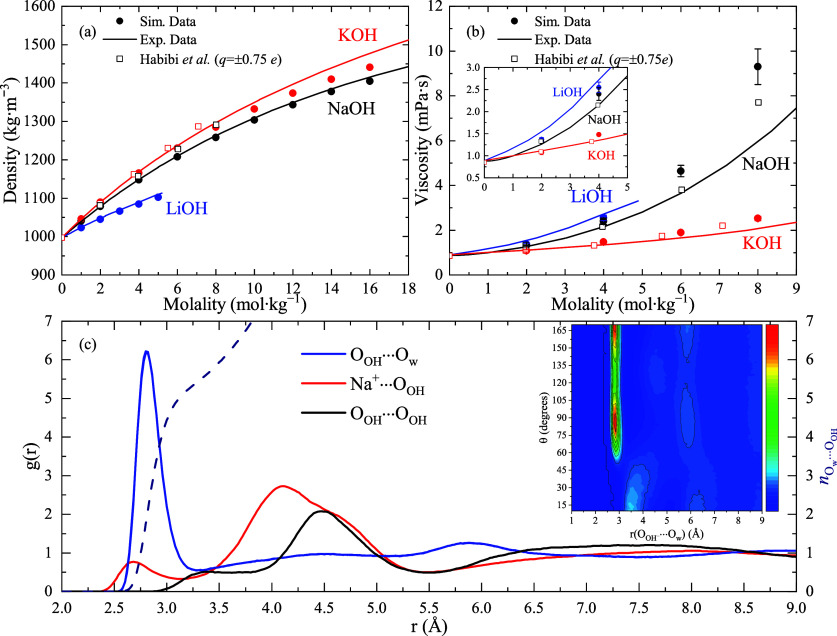
Experimental and simulated properties of different hydroxide solutions. (a) Densities as a function of the molality (error bars are smaller than the symbol size); and (b) Viscosities as a function of the molality, both at room temperature and pressure. Filled circles: Molecular Dynamics results were obtained using the force field developed here. Empty symbols: Simulations for the model with a net charge of ±0.75*e* developed by Habibi et al.^26^ Solid lines: Experimental results from ref (29). The inset shows the enlarged region of the graph for 0–5 *m*. (c) Site–site RDF for selected atom pairs of a 16 *m* NaOH solution at room pressure and temperature. The average number of water molecules around OH^–^ (*n*_*O*_*w*_···*O*_OH__) is plotted as a dashed line. The inset shows the 2D-contour plot of *g*(*r*_*O*_OH_···*O*_*w*__|θ), which is a combination of the *g*(*r*_*O*_OH_···*O*_*w*__) and the angular distribution function constructed with the angle θ = *∠H*_OH_*O*_OH_*O*_*w*_ as variable. The color bar varies from blue to red with increasing the combined probability, *P*(*r*_*O*_OH_···*O*_*w*__|θ), of finding a O_w_ atom at a certain position from the O_OH_ atom forming an angle θ.

An important aspect to be considered is whether the price paid for accurately predicting thermodynamic and dynamic properties is a poor description of the solution structure. In Figure 1(c) some site–site radial distribution functions (SS-RDF) for a NaOH solution at 16 *m* are presented. Similar figures for the rest of the salts and NaOH (1 *m*) are collected in the Supporting Information (SI). The main information extracted from these figures is summarized in Table 2, together with the experimental distance range for the SS-RDF peaks obtained from experiments.^17,31−33^ The O_OH_–O_w_ RDF bears a clear and intense peak at 2.8 Å and a structureless feature from 3.5 Å on that appears as a consequence of a progressive weakening of the short-range (local) order observed as a bulk-like structure. Some experimental data indicate that the peak in the RDF corresponding to the O_OH_···O_w_ distance is located ∼0.5 Å below the O_*w*_···O_w_ distance.^33,34^ In our simulations this effect is not observed, and both peaks are overlapping, in agreement with the more recent ref (4). In the structure of the OH^–^–cation RDF (red curve of Figure 1(c)), the regular allocation of OH^–^ is of solvent separated nature, in which the anion may form either one or two H-bonds with the Na^+^-coordinating water molecules. This effect is consistent with the interpretation given in refs (17 and 35) and is observed in the double peak structure of *g*(*r*_Na^+^···*O*_OH__) at ∼4.1 and ∼4.6 Å. Similar features are observed in KOH solutions. A peak at ∼2.7 Å grows with the salt concentration. Consequently, for both these salts, the number of contact ion-pairs, CIP_±_, increases moderately with concentration within the range obtained from simulations employed to interpret experimental structural data of NaOH solutions.^4,17^

**Table 2:** Structural Properties of OH^–^ Electrolyte Solutions at the Lowest, Intermediate, and Highest Molalities of Each Salt^a^

Salt	*m*/mol·kg^–1^	CIP_±_	CIP_OH_	HN_OH_	*d*_*O*_OH_···*O*_*w*__/Å	*d*_*O*_OH_···*O*_OH__^b^/Å
LiOH	1	0	0	5.5	2.8	4.5
	5	0	0	5.5	2.8	4.5
NaOH	1	0	0	5.5	2.8	4.5
	8	0.05	0	5.6	2.8	4.5
	16	0.3	0.12	5.5	2.8	3.4/4.5
KOH	1	0.1	0	5.5	2.8	4.5
	8	0.5	0	5.3	2.8	4.5
	16	1.1	0.05	4.5	2.8	3.4/4.4
OH^–^(exp.)	–	[0.1–1.45]^c^	[0.1–2.3]^c^	[3–5.5]^d^	[2.3–2.7]^d^	[3.3]/[4.1]^d^

a The selected features are the values of CIP_±_ (*i.e.*, cation and anion contacts) and CIP_OH_ (hydroxide–hydroxide contacts), the hydration numbers of the hydroxide anion (HN_OH_) and the positions of the first maximum of the OH^–^–O_w_ (*d*_*O*_OH_···*O*_*w*__), OH^–^–OH^–^ (*d*_*O*_OH_···*O*_OH__), and the counterion–OH^–^ (*d*_*CI*···*O*_OH__) in the corresponding radial distribution functions. The last line stands for the range of experimental data, when available. The discussion about the counterion–water structures can be found in refs (22 and 23).

b Both the contact distance (if appreciable), X, and the maximum of the SS-RDF, Y, are provided in the format X/Y.

c Taken from ref (17) for NaOH solutions. In this reference, the number of CIP are evaluated from the RDFs employed to simulate the X-ray scattering spectra.

d Taken from ref (26).

Finally, while the hydration number of OH^–^ is, in principle, higher than that observed from most experiments, in the inset of Figure 1(c) we present a 2D-contour plot of the combined *g*(*r*_*O*_OH_··· *O*_*w*__) RDF and the angular distribution function associated with the angle θ = *∠H*_OH_*O*_OH_*O*_*w*_ formed by the O_OH_–H_OH_ bond and the O_OH_···O_w_ distance. It is shown that at the maximum of the RDF, the maximum probability for the angle formed by the OH^–^ bond and the O_w_ atoms, θ, has peaks at ∼85° and ∼180°. Gathering this integrated information, a preferential (first-shell) hydration of the hydroxide ion in the vicinities of the O_OH_ atom can be inferred. This result is consistent with quantum chemical calculations (see ref (11)) and similar to the observations employing elaborated models.^36^ However, as pointed out in both experimental and computational studies in refs (37 and 38), it was found that the number of hydrogen bonds (HBs) per OH^–^ ion is four strong HBs (at distances smaller than 0.30 nm) and one weak HB (with distances higher than 0.30 nm and smaller than 0.35 nm), independent of the salt concentration. Nonetheless, the interactions in our model are not directional enough and provokes a higher HN_OH_ because of the less stringent distribution of the water coordination around the anion, but we also predict OH^–^···OH^–^ contacts, observed as a shoulder peaking at a distance of 3.4 Å in the corresponding RDF, in close agreement with that found in ref (17). Although the number of running contact anion–anion pairs predicted in our model is smaller than that reported, it should be emphasized, however, that this value is strongly sensitive to the upper integration limit, which is unclear in the original reference. The regular OH^–^···OH^–^ average distance peaks at ∼4.5 Å, a figure 0.4 Å higher than that reported in ref (17). On balance, given that the uncertainties of experimental data are typically ±0.2 Å, our Molecular Dynamics results for the structure can be considered of semiquantitative quality, even for the contraction trend of the water molecules coordinated in the second hydration shell as the salt concentration increases (see Figure S4).^33,34,39^

Subsequently, we have tested the performance of our model against the temperature of maximum density, TMD. Since this property hides information about the structural modifications provoked by ions added to water, its significance is springing up among experimental researchers.^40^ In the diluted regime, the TMD shifts (defined as Δ = TMD_*solution*_ – TMD_*water*_) can be rationalized assuming interionic interactions are negligible. Hence, a “group contribution” approach is widely used to evaluate the individual ion contribution (*K*_*m*_^±^) to the observed Despretz constant, *K*_*m*_ = lim_*m*→0_ Δ/*m*, i.e., *K*_*m*_ = ν_+_*K*_*m*_^+^ + ν_–_*K*_*m*_^–^ with ν_+_ and ν_–_ the stoichiometry coefficients of the cation and the anion, respectively. Surprisingly, we only found direct experimental values of the TMD for KOH in the dated ref (41) (although no data for the dependence of the density on the temperature is reported). Here, we performed experiments to evaluate the dependence of the density on the temperature for 1 *m* solutions of these salts (see Methods for details about the experimental procedure). These data are shown in Figure 2(a) together with the simulation results, which are collected in tabular form in the SI. The experiments lead to TMD_NaOH_^*exp*^ = 258.0 K and TMD_KOH_^*exp*^ = 259.4 K, in agreement with the simulations, from which TMD_NaOH_^*MD*^ = 257.1 K and TMD_KOH_^*MD*^ = 261.0 K, *i*.*e*., a deviation of −0.3% and −0.6%, respectively. The deviations of the density at the TMD are, at most, −0.2%. This observation constitutes a first indication of the transferability of the interaction parameters of the model in the captivating supercooled region, i.e., ∼40 K below the room temperature at which the parameters of the potential were optimized.

**Figure 2:**
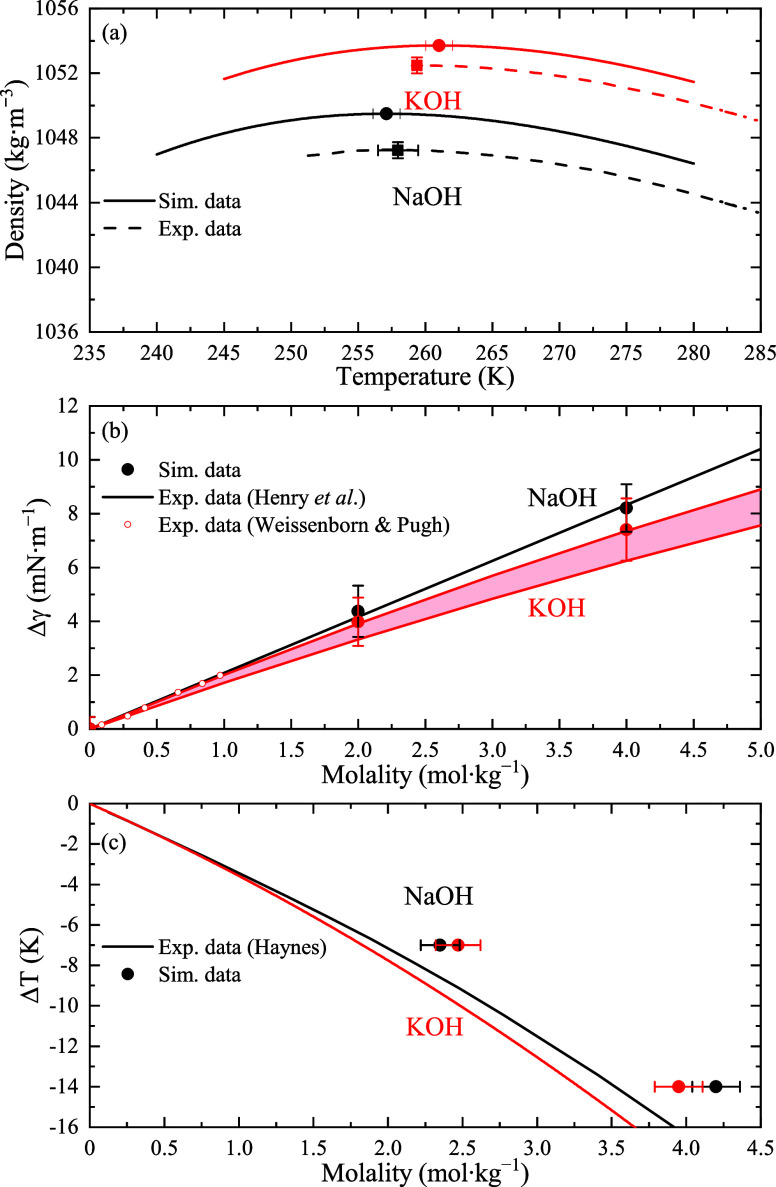
Experimental and simulated properties of different hydroxide solutions. (a) Simulated and experimental densities of 1 *m* solutions of NaOH and KOH as a function of the temperature. The continuous/dashed lines are cubic polynomial fits to the simulated/experimental data from which the TMD values are analytically extracted. Full squares and circles represent the experimental and simulated TMD values, respectively. (b) Values of Δγ = γ – γ_0_ for NaOH and KOH at 298 K. Full symbols stand for the simulation results, the shaded area enclosed by the continuous lines corresponds to the Δγ range extracted from the experimental derivatives *dγ*/*dm* collected in ref (43), and empty symbols are the experimental values of ref (44) for KOH solutions. (c) Freezing point depression of NaOH and KOH solutions. Lines stand for experimental data from ref (41) and symbols denote the simulation values.

Then, we extracted the individual ion contribution to the TMD. Consequently, using the experimental *K*_*m*_^±^ constants for K^+^ and Na^+^ derived by us in ref (42), these results lead to an average ion contribution to the Despretz constant of ⟨*K*_*m*_^OH^–^^ ⟩ = −8.2 K·mol·kg^–1^. From this ⟨*K*_*m*_^OH^–^^⟩, the values of the TMD can be derived as TMD_NaOH_^*group*^ = 257.3 K and TMD_KOH_^*group*^ = 260.1 K; that is, they are predicted with an accuracy better than 1 K.

The last parameters that we tested against the performance of our model are related to phase equilibrium. First, we will consider the liquid–vapor surface tension, γ, as a testing property for the reliability of the proposed force field. The experimental behavior of the surface tension of basic electrolytes indicates that, in the low concentration regime, it monotonously increases with the molality, with a rate *dγ*/*dm* slightly bigger for NaOH (2.08 mN·kg·m^–1^·mol^–1^) than for KOH (∼1.60 mN·kg·m^–1^·mol^–1^).^43^ The increase in γ and the relative slope values for these salts are indeed recovered by our model (see Figure S5), where the negative ion adsorption was estimated to be Γ_Na^+^_ ≈ Γ_OH^–^_ = −1.35 molecules·nm^–2^ and Γ_K^+^_ ≈ Γ_OH^–^_ = −0.8 molecules·nm^–2^ for 4 *m* solutions of NaOH and KOH, respectively (see SI for further details). Quantitatively, simulations carried out at 2 and 4 *m* demonstrated that the slopes *dγ*/*dm* are nicely captured by the model, with average values of *dγ*/*dm* ∼ 2.12 and 1.87 mN·kg·m^–1^·mol^–1^ for NaOH and KOH, respectively, in very good agreement with the experiments. Since there is a shift in the absolute value of γ for the TIP4P/2005 model as compared to experiments(∼3-4 mN·m when Lennard-Jones forces are not truncated), in Figure 2(b) we compare the Molecular Dynamics results for Δγ = γ – γ_0_, where γ_0_ is the surface tension of pure water, with those obtained from the experimental *d*Δγ/*dm* rates. Notice that for KOH the experimental data range reported in ref (43) is enclosed by a shaded region. The experimental data for KOH in the diluted (<1 *m*) regime,^44^ also included as small empty symbols in Figure 2(b), suggest that the upper limit is more reliable. Altogether, both Δγ and its first derivative with respect to *m* are excellently predicted by the Madrid-2019 force field here proposed, thus constituting a nice tool to study the riveting surface effects in OH^–^ solutions.^20^

As the icing on the cake, we have evaluated, via the direct coexistence method,^45^ the freezing temperature at different supercoolings, Δ*T* = *T*_*f*_^*sol*^ – *T*_*f*_^*w*^ (where *T*_*f*_^*w*^ is the freezing point of pure water and *T*_*f*_^*sol*^ is the freezing point of the solution). In Figure 2(c) the experimental values taken or extrapolated from ref (41) are presented together with simulation data. Deviations are found to be about 0.5 *m* for the highest supercooling considered here, i.e., Δ*T* = −14 K. Such deviations are previously reported in the recent ref (28) as typical for the Madrid-2019 model.

Heretofore, we have designed a force field for the important OH^–^ ion that leads to a remarkable agreement of the densities and viscosity coefficients of aqueous alkaline hydroxide solutions in a range of molalities. Taking into account that, after fixing atomic charges, the parametrization of Lennard-Jones crossing interactions is required to bypass the Lorentz–Berthelot rules, it might be reasonable to admit that, at least partly, polarizabilities and many-body effects arising from the coexistence of species of the type (H_2_O)_n_OH^–^ are *effectively* compensated by the appropriate combined selection of the pair potential parameters, regardless of their particular physical resemblance, at least in the dilute and intermediate concentration regimes. However, moderate deviations arose for even more concentrated solutions. The intensive search for appropriate interionic parameters was not successful in achieving a better match between experiments and simulations. The reason for this might presumably be behind the inadequate treatment of the water and counterion interactions with OH^–^ in the high concentration range. As commented, even when the experimental observation of a concentration- and counterion-dependent shrinkage in basic solutions^33,34,39^ is qualitatively reproduced in our simulations, this observation seems to indicate that a potential route to improve the parameters of the model involves modification of the Hamiltonian dealing with OH^–^–solvent interactions, that might be strictly dependent on the molality and/or on the counterion, as explicitly considered in ref (39). The idea of counterion-selective contact ion pairs (CIPs) has been recently recovered in refs (46 and 47) by using a combination of *ab initio* Molecular Dynamics simulations and vibrational spectroscopies. However, (*i*) even if the undertaking of finding state-dependent parameters for hydroxide can be laid out, this situation is not desirable from a thermodynamic perspective when derivatives of the thermodynamic potentials with respect to the composition are needed, i.e., for the calculation of the chemical potential, and (*ii*) opportunely, basic concentrations above those correctly predicted by the present force field are of scarce practical interest. A promising alternative route to overcome this feature and to provide a reactive model accounting for structural diffusion (in bulk or stringent geometries^48^) is the development of machine-learning-derived models.^49−52^ Since the accuracy of these models relies on the accuracy of the functional they derived from, there is room so far for improvements in this interesting research line.

In conclusion, we present a classical and nonpolarizable force field for the hydroxide ion to be used with the TIP4P/2005 water model. With the optimized parameters we have achieved reproduction not only of the dependence of the density and the viscosity coefficient on the concentration for LiOH, NaOH and KOH solutions in the low and intermediate concentration regimes but also of the TMD and equilibrium properties such as the surface tension and the freezing point depression. Hence, the force field proposed here allows one to extend the computational exploration of physical processes such as phase transitions or nucleation events in basic electrolytic solutions, significantly enlarging the applicability of the TIP4P/2005 and the Madrid-2019 force fields in these conditions.

## Methods

### Quantum Chemistry Calculations

Geometry optimizations were performed with the Gaussian16 software package^53^ using Density Functional Theory at the B3LYP/6-311++G(d,p) level. The wave function file seeded the Multiwfn software^54^ to evaluate the charge analysis within the ADCH approach.

### Molecular Dynamics Simulations

Molecular simulations were performed in the framework of the Molecular Dynamics method on a system comprised by 555 TIP4P/2005 water molecules and the necessary number of cations and anions to get the desired molality. Water–counterion and counterion–counterion parameters were chosen following refs (22 and 23). Unless otherwise mentioned, we employed the isothermal–isobaric (*NpT*) ensemble at 1 bar using the GROMACS 4.6.7 package^55^ to obtain the densities. Notice that selecting the molality as the concentration unit precludes changes in the concentration during the *NpT* simulations, in which the volume fluctuations also provoke concentration changes in the molarity scale. Temperature and pressure were kept constant employing the Nosé–Hoover thermostat and the isotropic Parrinello–Rahman barostat with a relaxation time of 2 ps, respectively. The Lagrangian LINCS algorithm was chosen to impose the holonomic constraints. The leapfrog algorithm with a time step of 2 fs was selected to integrate the equations of motion. A cutoff of 10 Å was set for both the excluded volume and electrostatic interactions, with the latter being treated within the Particle Mesh Ewald method. Long-range corrections for pressures and internal energies were also included for the Lennard-Jones interaction. The same methodology was followed to calculate the temperature of the maximum in density. Solubility tests were also performed for the highest concentration cases by computing the trajectories in boxes containing typically 4440 water molecules over 50 ns in the *NpT* ensemble. If a precipitation event occurred, the ion–ion interaction were tuned appropriately. Overall, the simulations ran between 40–1000 ns for each temperature and salt from previously equilibrated configurations.

Atom-to-atom RDF was also obtained in the *NpT* simulations. From these structural data we evaluated the hydration number (HN) and the number of contact ion pairs (CIPs) from the corresponding RDF. Particularly, cation–anion CIP (CIP_±_) is calculated using the number density of cations or anions, ρ_±_, as
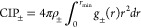
1where *g*_±_(*r*) is the cation–anion RDF and *r*_*min*_ the position of the first minimum of the RDF. The anion–anion CIP (CIP_OH_) is similarly defined as
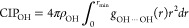
2where ρ_OH_ is the number density of the OH^–^ ion and *g*_OH···OH_(*r*) the OH^–^–OH^–^ RDF. Here, the position of *r*_*min*_ should be the minimum of *g*_OH···OH_(*r*). Finally, the hydration number of OH^–^ (HN) was evaluated as
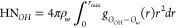
3where ρ_*w*_ is the number density of water and *g*_*O*_OH_···*O*_*w*__(*r*) the O_OH_–O_w_ RDF. Also, the number of HBs is evaluated according to the angle and distance criteria explained in ref (56).

Additionally, the transport properties were evaluated in the canonical (*NVT*) ensemble employing 4440 water molecules in an orthogonal box after a 20 ns equilibration in the *NpT* ensemble. Particularly, the diffusion coefficients were calculated from the mean-squared displacement (in a time-window avoiding subdiffusive effects) via the Einstein relation afterward corrected with the Yeh and Hummer prescription.^57^ Shear viscosities were calculated within the Green–Kubo formalism following the methodology reported in ref. (58). The surface tension is calculated in the *NVT* ensemble using the virial approach as in ref (28). Particularly, we employed a system of 6660 water molecules and the corresponding number of ions in contact with water vapor in an elongated box. Typically, *L*_*x*_ = *L*_*y*_ ≈ 3*L*_*z*_, and the cutoff was set to 1.4 nm. Finally, we studied the freezing point depression at different supercooling, using the direct coexistence method (see ref (45) for further details) at room pressure. Briefly, the secondary prismatic plane (12̅10) of a slab (2048 molecules) of ice I_h_ was put in contact with either NaOH or KOH aqueous solutions ∼1.8 *m* (65 × 2 ions and 2000 water molecules). The simulations were run for 1 μs for equilibration and 1 μs for production.

### Experimental Determination of the TMD

Solutions were made by weighing in an AE-240 balance, with an uncertainty of ±0.05 mg. Since both salts are hygroscopic, we performed an acid–base pH-metric titration, using oxalic acid as a primary standard, to obtain the mass fractions of NaOH (*w* = 0.991%) and KOH (*w* = 0.849%). The concentrations are further confirmed by the comparison between the reported^29^ and newly measured densities at 25 °C and 1 *m*. The difference was around a few tenths of kg·m^–3^, which is the experimental uncertainty.

Densities were determined using a homemade picnometer that consisted of a flask attached to a capillary tube. From the position of the meniscus inside this capillary *L*, the density of the sample can be easily found using
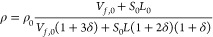
4where *V*_*f*,0_, *S*_0_ and *L*_0_ are the flask volume, the capillary cross-sectional area, and the meniscus position at the reference temperature *T*_0_, respectively, determined from calibration with pure water. δ = α(*T* – *T*_0_), where α the glass linear thermal expansivity accounts for the dilatation of the flask. Finally ρ_0_ is the density of the sample at the reference temperature, determined using a vibrating tube densimeter DMA 5000 from Anton Paar. Densities for NaOH at temperatures below −12 °C were determined using thin capillary tubes, with a very small volume to avoid freezing of the sample. Further details about these experimental techniques are reported elsewhere.^42^ Experimental uncertainty in density was calculated by identifying the main uncertainty sources. Briefly, the main contributions were the temperature, the meniscus height, and the parameters *V*_*f*,0_, *S*_0_, and α, the latter obtained from the calibration with pure water. The uncertainties of each source were evaluated by using statistical (type A uncertainty) or nonstatistical (type B) methods. Applying the standard procedure (see ref (59)), the total uncertainty budget is evaluated. The TMD was obtained from the ρ versus *T* fit by equating its derivative to zero. Therefore, it has a contribution easily estimated from the uncertainty of the fitting parameters and another one due to the uncertainty in temperature and density, which is the larger one, around 70% of the total uncertainty budget. Uncertainties were estimated as ±0.5 kg·m^–3^ for densities, ±0.3 K for the TMD of KOH, and ±1.5 K for the TMD of NaOH.

## Supporting Information Available

*Topology* file for GROMACS with the optimized potential parameters (PDF)
Raw simulated data for densities, viscosities, surface tension and temperature of maximum in density; Raw experimental densities against temperature; Representative site–site radial distribution functions, Comparison of the experimental surface tension with the raw simulated data for NaOH and KOH; Determination of the ion adsorption at the liquid–vapor interface for NaOH and KOH (PDF)
