# Supplementary material for: Dressing a Nonpolarizable Force Field for OH– in TIP4P/2005 Aqueous Solutions with Corrected Hirshfeld Charges
Source: J Phys Chem Lett. 2024 Sep 9;15(37):9411–8. doi: 10.1021/acs.jpclett.4c02261 (PMC11417996; doi:10.1021/acs.jpclett.4c02261)
Supplement: Supplementary file 1 — jz4c02261_si_001.pdf [file jz4c02261_si_001.pdf]

SUPPLEMENTARY MATERIAL

“Dressing a non-polarizable force field for OH^- in TIP4P/2005 aqueous solutions with corrected Hirshfeld charges”

Marcos de Lucas[1], Samuel Blazquez[1], Jacobo Troncoso[2], Carlos Vega[1], Francisco Gámez[1,*]

[1]Departamento de Química Física, Fac. Ciencias Químicas, Universidad Complutense de Madrid, 28040 Madrid, España.
[2]Departamento de Física Aplicada, Universidade de Vigo, Escola de Enxeñaría Aeronáutica e do Espazo, E 32004, Ourense, España

*Corresponding author: frgamez@ucm.es

```
[ defaults ]
; nbfunc      comb-rule     gen-pairs     fudgeLJ       fudgeQQ
 1            2             no            1.0     1.0

[atomtypes]
;name   mass    charge  ptype  sigma      epsilon
Na    22.9898     0.850     A  0.22173668   1.47235577
Mg    24.305      1.700     A  0.11629000   3.65190000
Ca    40.078      1.700     A  0.26656000   0.50720000
K     39.0983     0.850     A  0.23014000   1.98574000
Li    6.94100     0.850     A  0.14397000   0.43508986
Rb    85.4678     0.850     A  0.29949800   1.86231400
Cs    132.90545   0.850     A  0.35210130   0.37595960
F     18.998403   -0.850    A  0.37898200   0.03096370
Cl    35.4530     -0.850    A  0.46990563   0.07692308
Br    79.904      -0.850    A  0.48252500   0.11279500
I     126.90447   -0.850    A  0.50497500   0.17901000
OSO4  24.01565    -0.650    A  0.36500000   0.83740000
SSO4  0           0.900     D  0.35500000   1.04670000
IW    0           0.000     D  0.00000000   0.00000000
OWT4  15.9994     0.000     A  0.31589000   0.77490765
```

HW 1.00794 0.000 A 0.00000000 0.00000000

OHI 15.9994 0.000 A 0.34000000 0.25088977

HI 1.00794 0.000 A 0.14430000 0.18390827

[nonbond_params]

;i	j	func	sigma	epsilon
Cl	OWT4	1	0.42386698	0.06198347
Na	OWT4	1	0.26083754	0.79338830
Na	Cl	1	0.30051231	1.43889423
Mg	OWT4	1	0.18100000	12.0000000
Mg	Cl	1	0.30000000	3.00000000
Ca	OWT4	1	0.24000000	7.25000000
Ca	Cl	1	0.31500000	1.00000000
K	OWT4	1	0.28904000	1.40043000
K	Cl	1	0.33970000	1.40000000
Li	OWT4	1	0.21200000	0.70065003
Li	Cl	1	0.27000000	1.28294385
Rb	OWT4	1	0.35435000	0.10000000
Rb	Cl	1	0.39964200	0.34064100
Cs	OWT4	1	0.36629000	0.10000000
Cs	Cl	1	0.43185400	0.16155580
Br	OWT4	1	0.41985000	0.10000000
Li	Br	1	0.26145000	0.19937800
Na	Br	1	0.33850000	0.35677000
K	Br	1	0.37987900	0.42594000
Rb	Br	1	0.39172500	0.45832300
Cs	Br	1	0.43340800	0.19563200
Mg	Br	1	0.26551900	0.64180700
Ca	Br	1	0.36705200	0.23918500
I	OWT4	1	0.43495000	0.10000000
Li	I	1	0.32047000	0.27349800

Na I 1 0.36465800 0.51338700

K I 1 0.40055000 0.53659000

Rb I 1 0.41028800 0.51964600

Cs I 1 0.44379000 0.24645200

Mg I 1 0.28270700 0.80853400

Ca I 1 0.39418100 0.30132000

F OWT4 1 0.37745000 0.10000000

Li F 1 0.28454000 0.11026550

K F 1 0.34625000 0.22316700

Rb F 1 0.35725000 0.21612020

Cs F 1 0.39455000 0.09710500

OSO4 OWT4 1 0.34044500 0.62900000

Mg OSO4 1 0.24064500 2.74874271

K OSO4 1 0.32000000 1.28951878

Li OSO4 1 0.28448500 0.80360935

Rb OSO4 1 0.34000000 1.25080012

OHI OWT4 1 0.34500000 0.44092300

OHI Li 1 0.26448500 0.33039270

OHI Na 1 0.30336834 0.60778203

OHI K 1 0.32000000 0.70581330

[moleculetype]

; name nrexcl

water 1

[atoms]

; nr type resnr residu atom cgnr charge

1 OWT4 1 water OW1 1 0 15.9994

2 HW 1 water HW2 1 0.5564 1.00794

3 HW 1 water HW3 1 0.5564 1.00794

4 IW 1 water MW4 1 -1.1128 0.0

```
[constraints]
;i j funct doh  dhh
1     2     1     0.09572
1     3     1     0.09572
2     3     1     0.15139

[exclusions]
1     2     3     4
2     1     3       4
3     1     2     4
4     1     2     3

; The position of the dummy is computed as follows:
;
;                   O
;
;                   D
;
;         H                 H
;
; const = distance (OD) / [ cos (angle(DOH))    * distance (OH) ]
;           0.015 nm       / [ cos (52.26 deg)       * 0.09572 nm   ]

; Dummy pos x4 = x1 + a*(x2-x1) + b*(x3-x1)

[dummies3]
; Dummy from                        funct    a                    b
4     1     2     3     1     0.13193828     0.13193828
```

```
[moleculetype]
; name nrexcl
HO  1

[atoms]
; nr type resnr residu atom cgnr charge
1    OHI  1    HO   OHI  1    -1.0727    15.9994
2    HI   1    HO   HI   1     0.2227    1.00794

[constraints]
;i j funct doh  dhh
1      2      1      0.098

[exclusions]
1      2



[moleculetype]
; name nrexcl
Na  1

[atoms]
; nr type resnr residu atom cgnr charge
    1    Na     1    Na    Na      1    0.8500    22.9898

[moleculetype]
; name nrexcl
Cl  1

[atoms]
```

```
; nr type resnr residu atom cgnr charge
   1    Cl     1   Cl   Cl      1   -0.8500    35.453

[ moleculetype ]
; molname      nrexcl
Mg     1

[ atoms ]
; id    at type     res nr  residu name at name  cg nr  charge   mass
   1    Mg     1   Mg    Mg     1    1.7      24.3050

[ moleculetype ]
; molname      nrexcl
Ca     1

[ atoms ]
; id    at type     res nr  residu name at name  cg nr  charge   mass
1      Ca        1      Ca        Ca      1      1.7    40.078

[moleculetype]
; name nrexcl
K  1

[atoms]
; nr type resnr residu atom cgnr charge
   1    K      1   K    K      1    0.8500    39.0983

[moleculetype]
; name nrexcl
Li  1
```

[atoms]

; nr type resnr residu atom cgnr charge

1 Li 1 Li Li 1 0.8500 6.94100

[moleculetype]

; name nrexcl

Rb 1

[atoms]

; nr type resnr residu atom cgnr charge

1 Rb 1 Rb Rb 1 0.8500 85.4678

[moleculetype]

; name nrexcl

Cs 1

[atoms]

; nr type resnr residu atom cgnr charge

1 Cs 1 Cs Cs 1 0.8500 132.90545

[moleculetype]

; name nrexcl

F 1

[atoms]

; nr type resnr residu atom cgnr charge

1 F 1 F F 1 -0.8500 18.998403

[moleculetype]

; name nrexcl

Br 1

```
[atoms]
; nr type resnr residu atom cgnr charge
   1    Br     1    Br    Br      1    -0.8500    79.904

[moleculetype]
; name nrexcl
I 1

[atoms]
; nr type resnr residu atom cgnr charge
   1    I      1    I    I      1     -0.8500    126.90447


[moleculetype]
; name nrexcl
SO4   1

[atoms]
;nr   type resnr residu atom cgnr  charge
1    OSO4 1    SO4   O1  1   -0.65     24.01565
2    OSO4 1    SO4   O2  1   -0.65     24.01565
3    OSO4 1    SO4   O3  1   -0.65     24.01565
4    OSO4 1    SO4   O4  1   -0.65     24.01565
5    SSO4 1    SO4   S5  1    0.9       0.0

[constraints]
;i     j    funct    dist
; dist_OX=0.149; dist_XX=2*sqrt(2/3)*dist_OX
1      2      1      0.243316
1      3      1      0.243316
```

1	4	1	0.243316
2	3	1	0.243316
2	4	1	0.243316
3	4	1	0.243316

[exclusions]

1	2	3	4	5
2	1	3	4	5
3	1	2	4	5
4	1	2	3	5
5	1	2	3	4

[virtual_sitesn]

; Site funct from

5 1 1 2 3 4

[system]

water_sal

[molecules]

water 555

K 10

HO 10
