# Supplementary material for: Dressing a Nonpolarizable Force Field for OH– in TIP4P/2005 Aqueous Solutions with Corrected Hirshfeld Charges
Source: J Phys Chem Lett. 2024 Sep 9;15(37):9411–8. doi: 10.1021/acs.jpclett.4c02261 (PMC11417996; doi:10.1021/acs.jpclett.4c02261)
Supplement: Supplementary file 2 — jz4c02261_si_002.pdf [file jz4c02261_si_002.pdf]

SUPPLEMENTARY MATERIAL

"Dressing a non-polarizable force field for OH^- in TIP4P/2005 aqueous solutions with corrected Hirshfeld charges"

Marcos de Lucas[1], Samuel Blazquez[1], Jacobo Troncoso[2], Carlos Vega[1], Francisco Gámez[1,*]

[1]Departamento de Química Física, Fac. Ciencias Químicas, Universidad Complutense de Madrid, 28040 Madrid, España.
[2]Departamento de Física Aplicada, Universidade de Vigo, Escola de Enxeñaría Aeronáutica e do Espazo, E 32004, Ourense, España

*Corresponding author: frgamez@ucm.es

The Supplementary Material for the publication '*Dressing a non-polarizable force field for OH^- in TIP4P/2005 aqueous solutions with corrected Hirshfeld charges"* contains the experimental and simulations details in addition to the compilation of the numerical (raw data) information of the simulation results of all hydroxide salts considered in the main body of this work.

1. Raw Data

Table S1. Simulated densities as a function of the molality of LiOH, NaOH and KOH at 298 K and 1 bar.

LiOH		NaOH		KOH	
m (mol/kg)	ρ (kg/m^3)	m (mol/kg)	ρ (kg/m^3)	m (mol/kg)	ρ (kg/m^3)
1	1022.72	1	1040.39	1	1046.04
2	1045.25	2	1078.89	2	1089.71
3	1065.69	4	1147.69	4	1165.65
4	1084.55	6	1207.06	6	1229.88
5	1102.07	8	1258.35	8	1284.99
		10	1303.48	10	1332.12
		12	1342.84	12	1373.12
		14	1377.61	14	1409.3
		16	1404.47	16	1440.74

Table S2. Simulated viscosities as a function of the molality of LiOH, NaOH and KOH at 298 K and 1 bar.

LiOH		NaOH		KOH	
m (mol/kg)	η (mPa·s)	m (mol/kg)	η (mPa·s)	m (mol/kg)	η (mPa·s)
2	1.37	1	1.32	1	1.08
4	2.55	2	2.40	2	1.48
6		4	4.64	4	1.89
8		6	9.30	6	2.52

Table S3. Simulated surface tension as a function of the molality NaOH and KOH solutions at 298 K and 1 bar.

NaOH		KOH	
m (mol/kg)	γ (mN/m)	m (mol/kg)	γ (mN/m)
2	4.4	2	4.0

4	8.2	4	7.4

Table S4. Simulated densities as a function of the temperature of NaOH and KOH solutions 1 *m* at 1 bar.

NaOH		KOH	
T (K)	ρ (kg/m^3)	T (K)	ρ (kg/m^3)
240	1046.97	245	1051.60
245	1048.30	250	1052.84
250	1049.05	255	1053.43
255	1049.5	260	1053.66
260	1049.43	265	1053.56
265	1049.03	270	1053.23
270	1048.38	275	1052.45
275	1047.55	280	1051.44

Table S5. Experimental densities as a function of the temperature of NaOH and KOH solutions 1 *m* at 1 bar.

NaOH		KOH	
T (K)	ρ (kg/m^3)	T (K)	ρ (kg/m^3)
283.15	1043.7781	283.15	1049.4548
278.15	1044.9441	278.149	1050.5354
273.651	1045.8189	273.65	1051.3227
272.65	1045.9770	273.15	1051.3941
271.65	1046.1328	272.15	1051.5400
270.65	1046.2783	271.15	1051.6824
269.65	1046.4110	270.15	1051.8072
268.65	1046.5411	269.15	1051.9240
267.65	1046.6583	268.15	1052.0286
266.65	1046.7592	267.15	1052.1204
265.65	1046.8553	266.15	1052.2091
265.15	1046.8960	265.65	1052.2496
264.65	1046.9391	265.15	1052.2829
264.15	1046.9787	264.65	1052.3151
263.65	1047.0151	264.15	1052.3453
263.15	1047.0505	263.65	1052.3722
262.65	1047.0833	263.15	1052.3966
262.15	1047.1102	262.65	1052.4165
261.65	1047.1357	262.15	1052.4357
261.15	1047.1559	261.65	1052.4505
260.15	1047.2524	261.15	1052.4633
259.15	1047.2231	260.65	1052.4729
258.15	1047.2571	260.15	1052.479
257.15	1047.2658	259.65	1052.4818
256.15	1047.2463	259.15	1052.4818
255.15	1047.2198		
254.15	1047.1822		
253.15	1047.0543		
252.15	1046.9579		
251.15	1046.8979		

2. Site-site radial distribution functions.

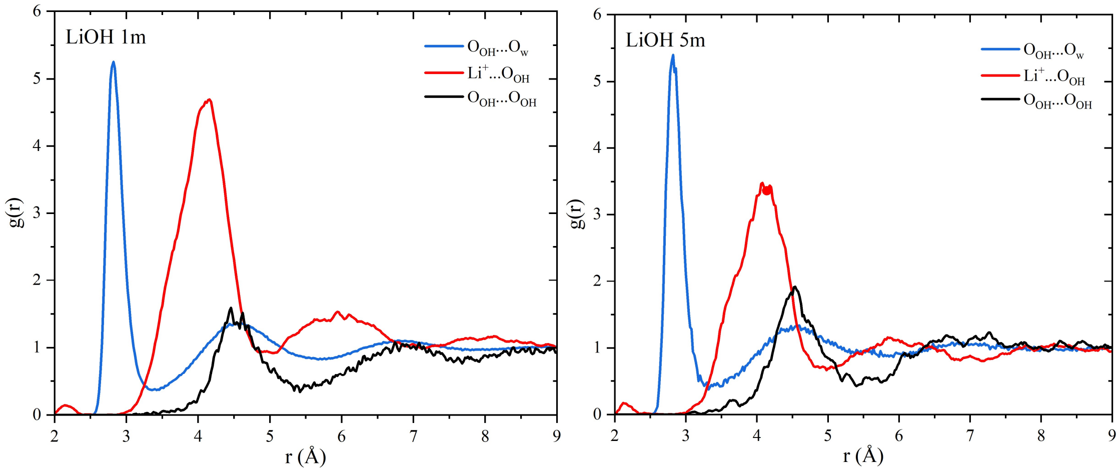

Figure S1. Site–site RDF for selected atom pairs of 1 *m* and 16 *m* LiOH solutions at room pressure and temperature.

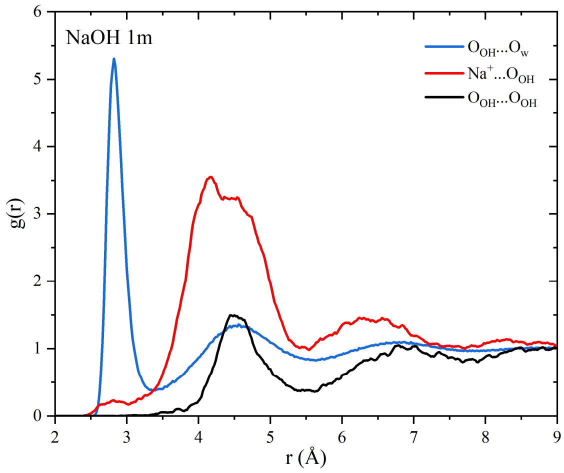

Figure S2. Site–site RDF for selected atom pairs of 1 *m* NaOH solution at room pressure and temperature.

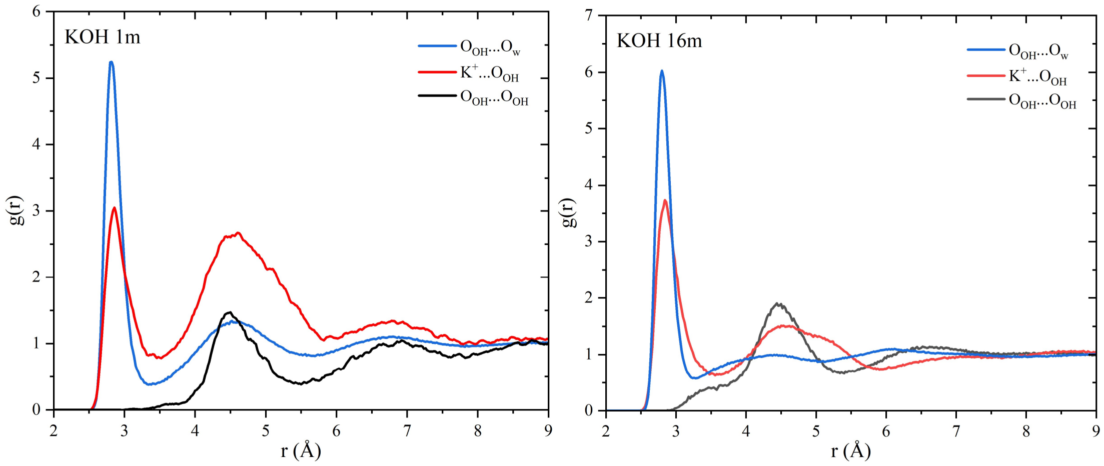

Figure S3. Site–site RDF for selected atom pairs of 1 *m* and 16 *m* KOH solution at room pressure and temperature.

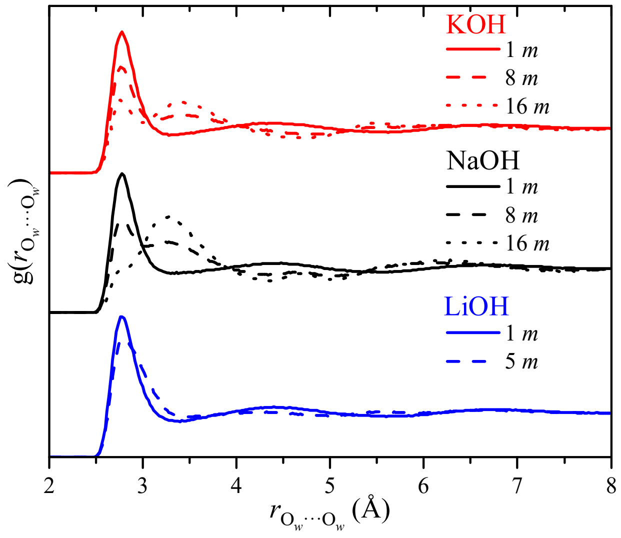

Figure S4. Site–site RDF for $O_w \cdots O_w$ pair for a selected set of concentrations for LiOH, NaOH, KOH solutions at room pressure and temperature.

3. Surface tension.

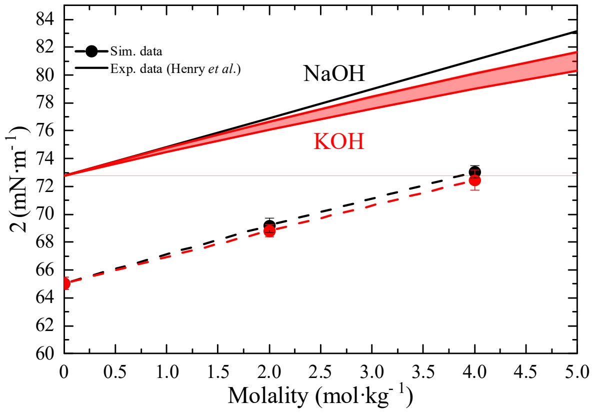

Figure S5. Raw simulation data of the surface tension against experimental data from Ref.[1]. Notice that since no long-range corrections have been applied, the surface tension is shifted down by about 3 mN/m according to Ref.[2].

[1] Henry, C. L.; Dalton, C. N.; Scruton, L.; Craig, V. S. Ion-Specific Coalescence of Bubbles in Mixed Electrolyte Solutions. J. Phys. Chem. C (2007), 111 (2), 1015–1023.

[2] de Miguel, M.; Vega, C. Surface tension of the most popular models of water by using the test-area simulation method. J. Chem. Phys. (2007), 126,154707.

4. Determination of the ion adsorption at the liquid-vapor interface.

To calculate the adsorption of Na^+, K^+ and OH^- at the liquid-vapor interface we used the calculated density profiles showed in Figure S1. First, we determined the position z_G of the so-called Gibbs dividing surface, *i.e.*, the surface that gives zero adsorption of water. Then, the adsorption of any species (β) at the water-vapor interface, Γ_β, can be calculated as follows:

$$\Gamma_\beta = \int_{-\infty}^{z_G} \left(\rho_\beta(z) - \rho_{\beta,H_2O}\right) dz + \int_{z_G}^{\infty} \left(\rho_\beta(z) - \rho_{\beta,vapor}\right) dz$$

where, $\rho_\beta(z)$ is the number density of species β along the z-axis and ρ_{β,H_2O} and $\rho_{\beta,vapor}$ are the average number densities of β in water and vapor phases respectively.

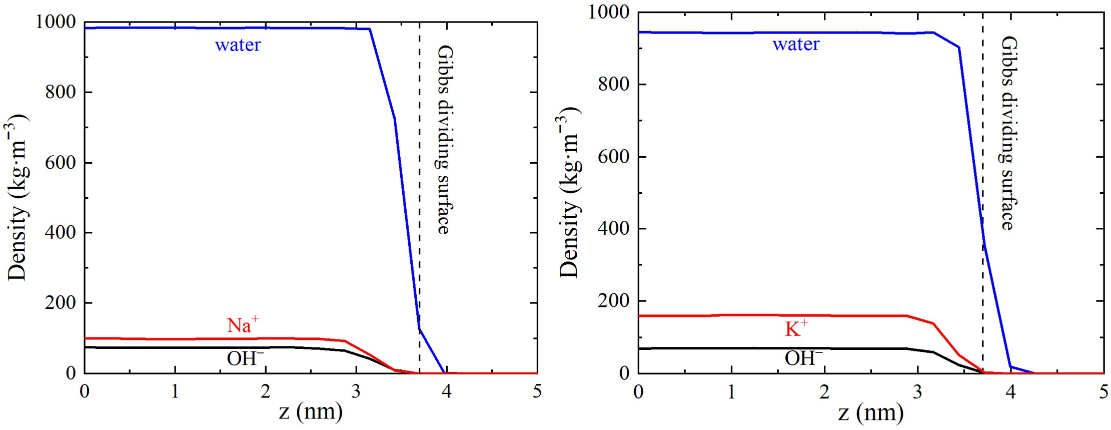

Figure S6. Density profiles of water (blue), OH^- (black) and counterion (red) for NaOH (right) and KOH (left) solutions 16 *m*. The position of the Gibbs dividing surface is also shown.
